# Neutrophil extracellular traps are induced in a psoriasis model of interleukin-36 receptor antagonist-deficient mice

**DOI:** 10.1038/s41598-020-76864-y

**Published:** 2020-11-19

**Authors:** Soichiro Watanabe, Yohei Iwata, Hidehiko Fukushima, Kenta Saito, Yoshihito Tanaka, Yurie Hasegawa, Masashi Akiyama, Kazumitsu Sugiura

**Affiliations:** 1 Department of Dermatology, Fujita Health University School of Medicine, 1-98 Kutsukake-cho, Toyoake, Aichi 470-1192 Japan; 2 Department of Dermatology, Nagoya University Graduate School of Medicine, 65 Tsurumai-cho, Showa-ku, Nagoya, Aichi 466-8550 Japan

**Keywords:** Drug discovery, Immunology, Medical research

## Abstract

Loss-of-function mutations in the interleukin (IL)-36 gene *IL36RN* are associated with psoriasis. The importance of neutrophil extracellular traps (NETs), web-like structures composed of neutrophil DNA, in the pathogenesis of psoriasis has been unclear. Here, we aimed to clarify the role of NET signaling in the deficiency of IL36 receptor antagonist (DITRA). We evaluated the severity of psoriasis-like lesions induced by imiquimod cream treatment in *Il36rn*^−/−^ mice. The mRNA levels of psoriasis-related cytokines were measured via real-time reverse transcription polymerase chain reaction, and the effects of Cl-amidine, a peptidyl arginine deiminase 4 (PAD4) inhibitor, on psoriasis-like lesions were evaluated. PAD4 is a histone-modifying enzyme that is involved in NET formation. Psoriasis area and severity index scores, epidermal thickness, and infiltrated neutrophil counts were significantly increased in *Il36rn*^−/−^ mice; NET formation was confirmed pathologically. Several cytokines and chemokines were upregulated in the skin lesions of *Il36rn*^−/−^ mice and Cl-amidine treatment improved these psoriasis-like lesions. These results suggest that NET formation plays an important role in the pathology of psoriasis-like lesions in these mice and might represent a promising therapeutic target for DITRA.

## Introduction

*IL36RN* encodes interleukin (IL)-36 receptor antagonist (IL-36Ra), an IL-1 cytokine family protein that strictly regulates IL-36 signaling. The IL-36 pathway is induced when IL-36α, β, or γ binds to its specific receptor, interleukin-1 receptor-related protein 2 (IL-1Rrp2), triggering the recruitment of the co-receptor IL-1 receptor accessory protein (IL-1RacP) and activation of the nuclear factor-kappa B (NF-κB), as well as mitogen-activated protein kinase signaling pathways. This enhances the transcription and secretion of pro-inflammatory cytokines^1,2^, which recruit neutrophils, T cells, and dendritic cells (DCs) in the skin. IL-36 stimulates the production of chemotactic agents for activated leukocytes, promoting leukocyte infiltration and acanthosis of the skin^3^. Loss-of-function mutations in *IL36RN* cause a recessively inherited autoinflammatory keratinization disease known as deficiency of IL-36Ra (DITRA)^4–9^. We previously prepared *Il36rn*^−/−^ mice and established a DITRA murine model by treating mice with a TLR4 agonist^10^, a severe contact hypersensitivity model by treatment with 1-fluoro-2,4-dinitorobenzene^11^, and a delayed cutaneous wound healing model^12^. It has been known that deficiency of IL36Ra induces severe epidermal proliferation and neutrophil infiltration in imiquimod (IMQ)-induced psoriasis-like lesions^13,14^.

The roles of T cells, macrophages, and DCs have been reported in the pathogenesis of psoriasis vulgaris^15–19^. However, the role of neutrophils in this pathology have gradually been clarified^20^ but have not been sufficiently elucidated. Neutrophils play important roles in many diseases, including infectious and neoplastic, autoimmune, and chronic inflammatory diseases^21–25^. Upon activation, neutrophils undergo a cell death process known as NETosis, in which nuclear substances are extruded into the extracellular space^26,27^. These structures are named neutrophil extracellular traps (NETs) and are large, web-like structures composed of granule proteins, histones, and decondensed DNA^28^. The existence of NETs has been reported in psoriatic skin, where they might play a role in inducing increased expression of human β-defensin-2^29^. Therefore, NETs and neutrophils can induce inflammation through various mechanisms, including inflammasome activation^30^, the triggering of Toll-like receptor 7 (TLR7) and TLR9 via self-antigen complexes such as cathelicidin antimicrobial peptide LL37 DNA^31^, macrophage pyroptosis stimulation^32^, and IL-36 cytokine processing and activation^33,34^.

Here, we aimed to clarify the role of NET signaling in DITRA and develop an effective therapy for IMQ-induced severe psoriasis in *Il36rn*^−/−^ mice. As NETs might be involved in DITRA, it could be possible to prevent the development of severe psoriasis-like lesions by prohibiting their formation. Therefore, this study was conducted to determine the immunological pathology associated with severe psoriasis-like lesions in mice with DITRA.

## Results

### Estimation of psoriasis area and severity index (PASI) scores and histological characteristics in ***Il36rn***^***−/−***^mice after consecutive topical application of IMQ cream for 3 days

**Figure 1:**
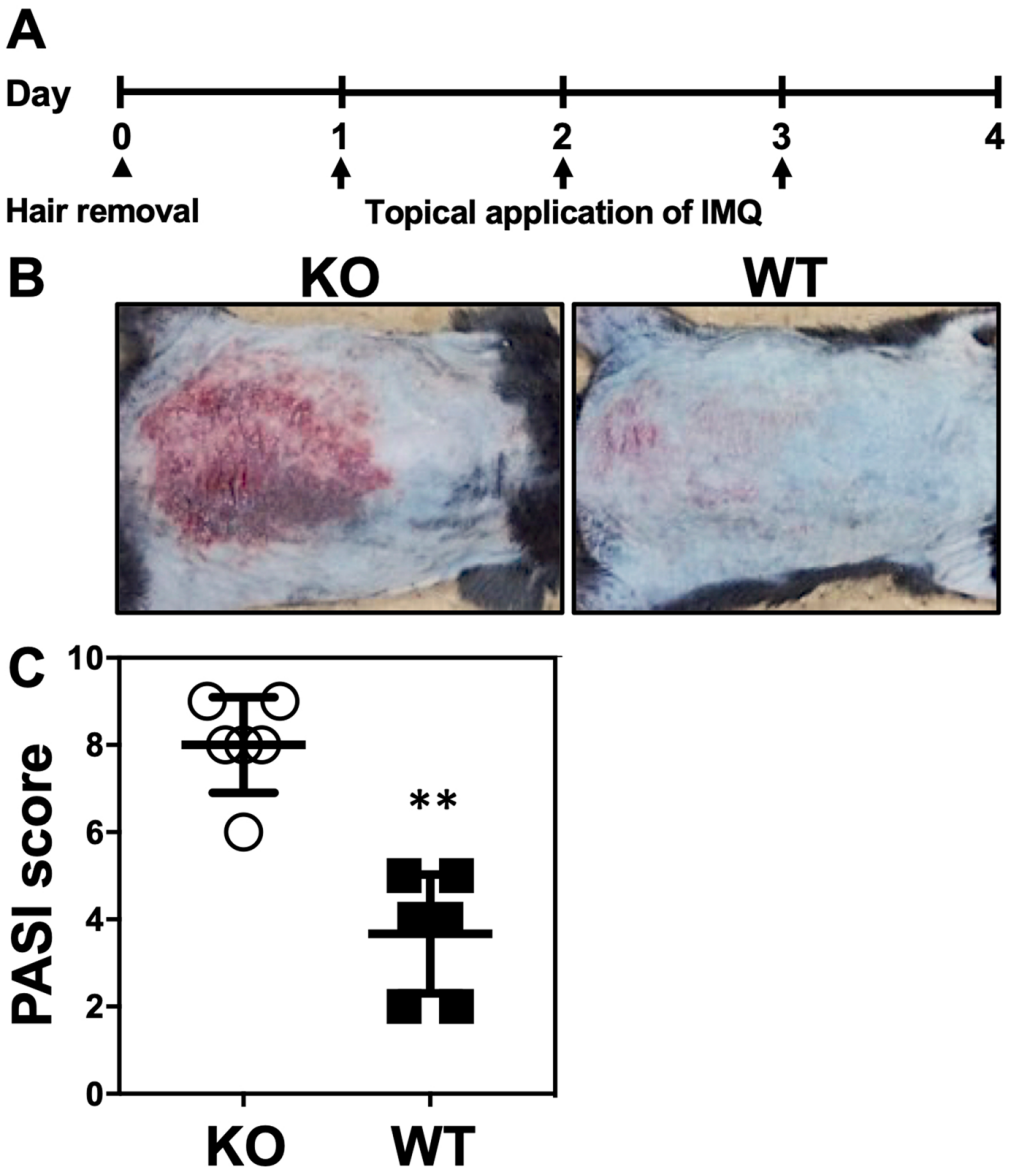
Imiquimod (IMQ) treatment induces psoriasis-like lesions in *Il36rn*^−/−^ mice. (**A**) Protocols for the development of IMQ-induced contact psoriasis-like lesions; (**B**) representative clinical images of back skin (KO: *Il36rn*^−/−^ mice, WT: wild-type mice). (**C**) Psoriasis area and severity index (PASI) scores of skin lesions in IMQ-treated mice on day 4 (n = 6). **p < 0.01.

**Figure 2:**
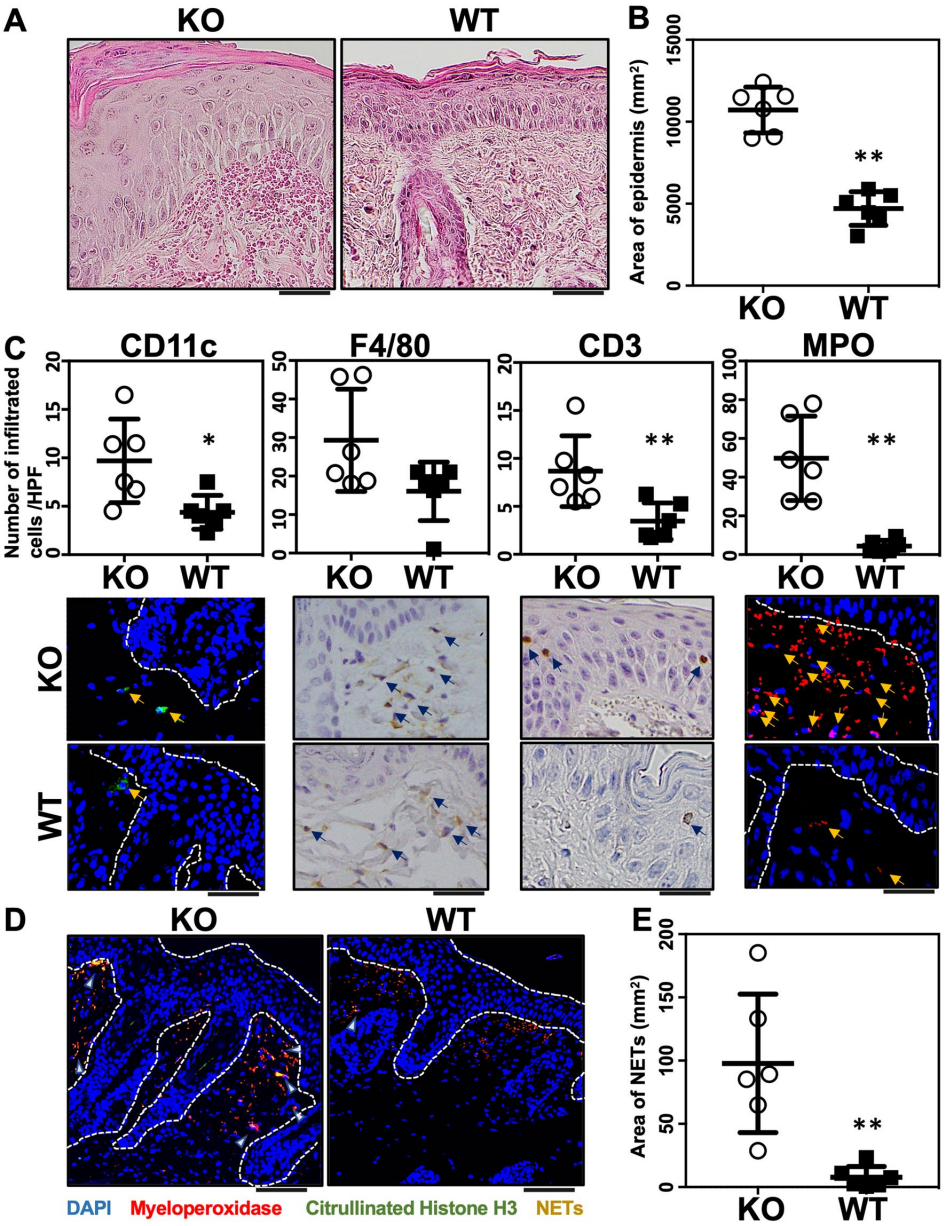
Area of epidermis, numbers of infiltrated neutrophils and neutrophil extracellular trap (NET) formation. (**A**) Histological images of skin sections from WT and *Il36rn*^−/−^ mice 3 days after topical imiquimod (IMQ) application. Scale bars, 100 µm. (**B**) The epidermal area within a distance of 10 mm was measured using ImageJ software (n = 6 animals/group; **p < 0.01 versus WT). (**C**) Numbers of infiltrated cells/high-power fields in back skin cross-sections from IMQ-treated *Il36rn*^−/−^ and WT mice (100 × magnification, nine sections per mouse, n = 6 animals/group; * p < 0.05, **p < 0.01 versus WT). Representative photos of CD11c, F4/80, CD3, and MPO. Arrow shows positive cells. (**D**) Enhanced NETosis (arrows) in the dermis of IMQ-treated *Il36rn*^−/−^ mice. Nuclear staining: 4′,6-diamidino-2-phenylindole (blue). Representative immunofluorescence image of NET structures stained with myeloperoxidase (red) and citrullinated histone (green). Scale bar, 50 μm. (**E**) Area of NETs/high-power fields in back skin cross-sections from IMQ-treated *Il36rn*^−/−^ and WT mice (40 × magnification, nine sections per mouse, Mann–Whitney *U* test, n = 6 animals/group; **p < 0.01 versus WT).

IMQ treatment for 3 consecutive days induced psoriasis-like lesions in *Il36rn*^−/−^ mice. For the purpose of assessing the severity of this psoriasis-like lesion, we evaluated the eruption using the PASI score^35^ and compared *Il36rn*^−/−^ and wild-type mice (n = 6). *Il36rn*^−/−^ mice showed a significant increase in PASI scores (Fig. 1A–C). Severe scaling was observed in IMQ-induced *Il36rn*^−/−^ mice. Next, we pathologically estimated epidermal area, the number of infiltrated neutrophils and NET formation. *Il36rn*^−/−^ mice showed a significant increase in the epidermal area (Fig. 2A,B) compared to that in wild-type mice after IMQ treatment for 3 consecutive days. To evaluate inflammatory cell infiltration in IMQ-induced psoriasis lesions, immunostaining for CD11c, F4/80, CD3, and myeloperoxidase (MPO) was performed. *Il36rn*^−/−^ mice showed a significant increase in CD11c-, CD3-, and MPO-positive cells compared to those in wild-type mice (Fig. 2C). In addition, to evaluate NET formation in IMQ-induced psoriasis-like lesions, immunofluorescent co-staining for MPO and citrullinated histone H3 was performed. *Il36rn*^−/−^ mice showed a significant increase in the area of NETs compared to that in wild-type mice (Fig. 2D,E). Thus, *Il36rn*^−/−^ mice developed severe IMQ-induced psoriasis-like lesions, increased infiltration of inflammatory cells, and increased NET formation compared to those in wild-type mice.

### Chemokine and cytokine expression in psoriasis-like lesions in *Il36rn*^−/−^mice

**Figure 3:**
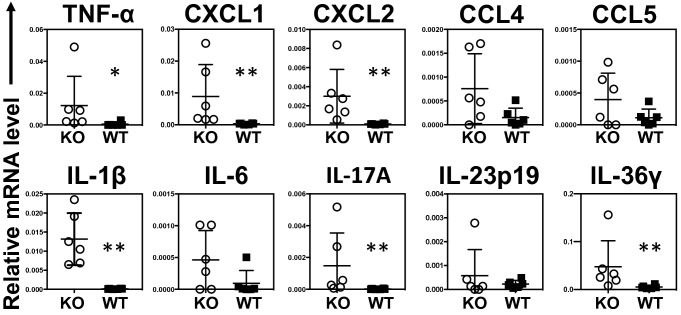
Skin mRNA expression levels. Real-time reverse transcription polymerase chain reaction (RT-PCR) analyses of psoriasis-related cytokines and chemokines from mouse skin samples (Mann–Whitney *U* test, n = 6 animals/group *p < 0.05, **p < 0.01 versus WT). Internal control: glyceraldehyde-3-phosphate dehydrogenase (*GAPDH*) mRNA. KO, IMQ-treated *Il36rn*^−/−^ mice; WT, IMQ-treated WT mice.

The expression of genes encoding tumor necrosis factor (TNF)-α, C–C motif chemokine ligand 4 (CCL4), CCL5, C-X-C motif chemokine ligand 1 (CXCL1), CXCL2, IL-1β, IL-6, IL-17A, IL-23p19, and IL-36γ after IMQ treatment for 3 consecutive days was measured via real-time reverse transcription polymerase chain reaction (RT-PCR) in *Il36rn*^−/−^ and wild-type mice (Fig. 3). *Il36rn*^−/−^ mice showed significantly increased *TNF-α*, *CXCL1*, *CXCL2*, *IL-1β*, *IL-17A*, and *IL-36γ* expression levels compared to those in wild-type mice. In contrast, the loss of IL-36Ra did not reach significant difference in a significant difference in *CCL4*, *CCL5*, *IL-6*, or *IL-23p19* mRNA expression levels in comparison to those in wild-type mice.

### Cl-amidine treatment of IMQ-induced psoriasis in ***Il36rn***^***−/−***^ mice

**Figure 4:**
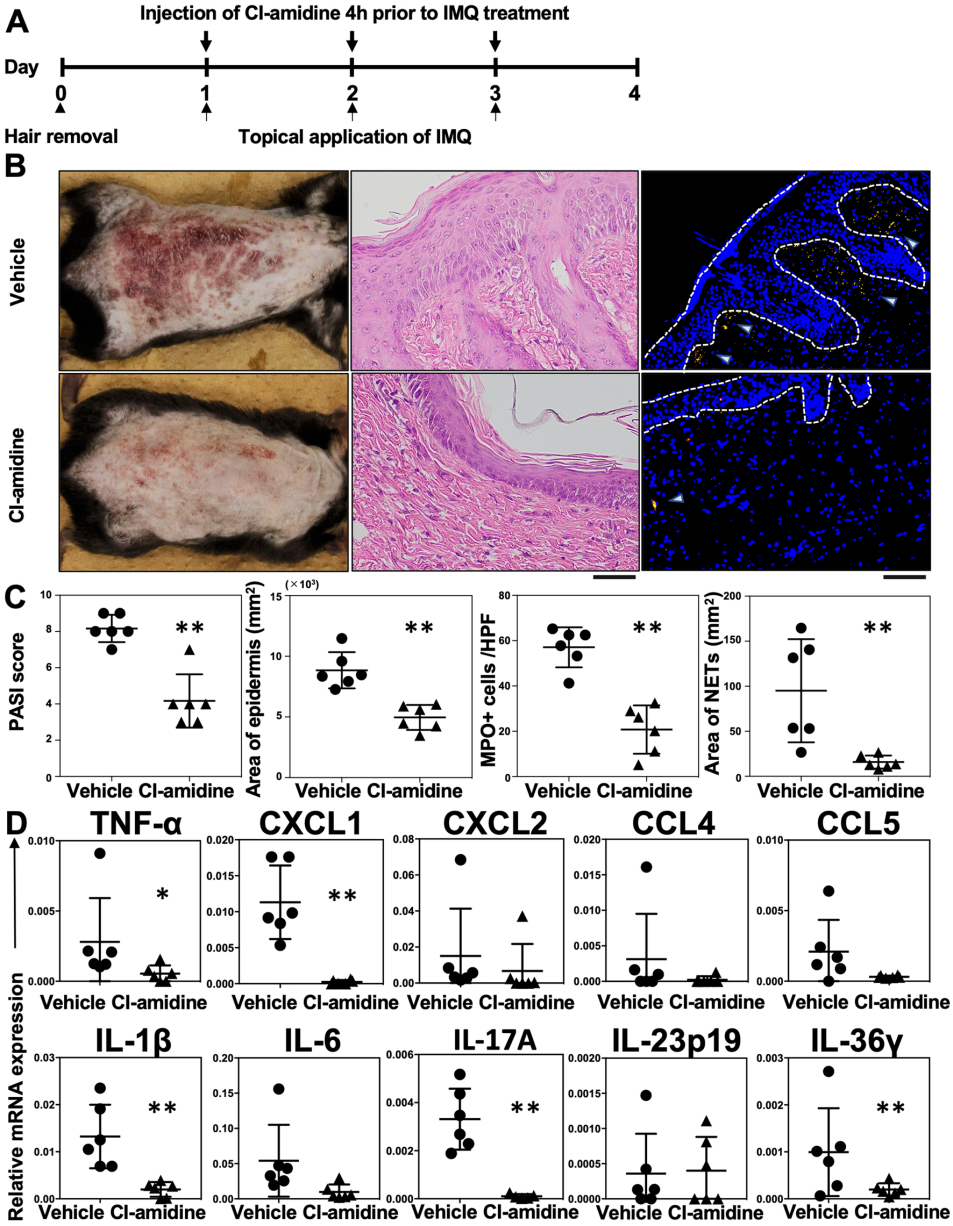
Subcutaneous injection of Cl-amidine decreases psoriasis-like lesions in *Il36rn*^−/−^ mice. (**A**) Protocols for the development of imiquimod (IMQ)-induced psoriasis-like lesions. For prevention experiments, *Il36rn*^−/−^ mice were subcutaneously injected with Cl-amidine (10 mg/kg) 4 h before IMQ treatment (days 1–3). (**B**) Representative clinical images of back skin and histological images of skin sections from *Il36rn*^−/−^ mice treated with Cl-amidine or vehicle 3 days after IMQ challenge. Scale bars, 100 µm. (**C**) Left: psoriasis area and severity index (PASI) scores of skin lesions in IMQ-challenged mice treated with Cl-amidine or vehicle on day 4 (Mann–Whitney *U* test, n = 6 animals/group; **p < 0. 01 versus vehicle-treated *Il36rn*^−/−^ mice). Second from left: the area of the epidermis within a distance of 10 mm was measured using ImageJ software. Third from left: number of MPO^+^ cells/high-power field in back skin cross-sections from Cl-amidine- or vehicle-treated *Il36rn*^−/−^ mice (100 × magnification, nine sections per mouse, Mann–Whitney *U* test, n = 6 animals/group; **p < 0.05 versus vehicle-treated *Il36rn*^−/−^ mice). Right: area of NETs/high-power field in back skin cross-sections from Cl-amidine- or vehicle-treated *Il36rn*^−/−^ mice (40 × magnification, nine sections per mouse, Mann–Whitney *U* test, n = 6 animals/group; **p < 0.05 versus vehicle-treated *Il36rn*^-/-^ mice). (**D**) Real time RT-PCR analyses of psoriasis-related cytokines and chemokines from mouse skin samples (Mann–Whitney *U* test, n = 6 animals/group *p < 0.05, **p < 0.01 versus vehicle-treated *Il36rn*^−/−^ mice). Data were expressed relative to *GAPDH* levels.

NET formation was enhanced in *Il36rn*^−/−^ mice. Therefore, we hypothesized that NETs would be involved in the pathogenesis of IMQ-induced psoriasis-like lesions. It has been known that Cl-amidine, a pan-peptidyl arginine deaminase (PAD) inhibitor, suppresses NET formation by inhibiting PAD4, an enzyme necessary for NET formation^36–39^. The effects of Cl-amidine were investigated in IMQ-induced psoriasis-like lesions in *Il36rn*^−/−^ mice. Cl-amidine (10 mg/kg/day) or the same amount of vehicle was subcutaneously administered for 3 days (day 1–3), 4 h prior to IMQ treatment (Fig. 4A). Interestingly, PASI scores, epidermal area, the number of infiltrated MPO-positive cells, and the area of NETs were significantly reduced by Cl-amidine administration compared with those measured in vehicle-treated *Il36rn*^−/−^ mice (Fig. 4B,C). In addition, *TNF-α*, *CXCL1*, *IL-1β*, *IL-17A*, and *IL-36γ* mRNA expression levels in IMQ-induced psoriasis-like lesions were significantly reduced by Cl-amidine administration compared to those in vehicle-treated *Il36rn*^−/−^ mice (Fig. 4D). CXCL2 (*p* = 0.0628), CCL5 (*p* = 0.0649), and IL-6 (*p* = 0.0648) levels tended to be decreased by Cl-amidine administration compared to those in vehicle-treated *Il36rn*^−/−^ mice (Fig. 4D). However, there were no significant differences in CCL4 and IL-23p19 expression levels between the Cl-amidine treatment and control groups (Fig. 4D). Thus, blockade of NETosis by Cl-amidine suppressed severe IMQ-induced psoriasis-like lesions in *Il36rn*^−/−^ mice.

### In vitro macrophage and NET culture

In our study, *Il36rn*^−/−^ mice showed significantly increased IL-36γ expression levels compared to those in wild-type mice (Fig. 3). It has been reported that NETs stimulate monocytes and induce secretion of IL-1β, IL-6, IL-23, and TNF-α^40^. In addition, the activation of monocytes leads to cell maturation with an increase in Th17 cells, leading to IL-36γ production by keratinocytes through T-cell mediated immune reactions via the IL-17/IL-23/IL-22 axis^40,41^. Therefore, it is possible that monocytes activated by NETs induce the secretion of IL-36γ through T-cell mediated immune reactions via the IL-17/IL-23/IL-22 axis. However, it has been reported that macrophages also play an important role in the pathogenesis of psoriasis^19^ and that NETs stimulate IL-1β and IL-18 release by macrophages derived from lupus patients^30^. In addition, it has been reported that NETs promote peritoneal macrophage pyroptosis, a caspase-1-dependent regulated cell death, and the production of macrophage-derived Il-1β and TNF-α^32^ in sepsis. Furthermore, it is also known that IL-36 produced by macrophages is involved in the pathological condition in psoriasis, rheumatoid arthritis, and Crohn’s disease^42^. Therefore, we hypothesized that the increase of IL-36γ in *Il36rn*^−/−^ mice is produced by macrophages activated by NETs. To test this possibility, we performed co-culture of macrophages with NETs, and measured mRNA expression levels of *TNF-α*, *IL-1β*, and *IL-36γ*, which are involved in the pathological condition of psoriasis.

**Figure 5:**
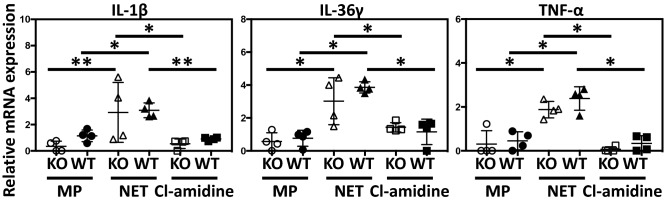
NETs promote the production of psoriasis-related cytokines from macrophages. Real-time RT-PCR analyses of psoriasis-related cytokines from mouse peritoneal macrophages (Mann–Whitney *U* test, n = 4 plates/group *p < 0.05, **p < 0.01). MP: macrophage, NET: macrophage incubated with NETs, Cl-amidine: macrophage incubated with NETs pretreated with Cl-amidine. Data were expressed relative to *GAPDH* levels.

Peritoneal macrophages of wild-type mice and *Il36rn*^−/−^ mice were used for in vitro analysis. *IL-1β*, *IL-36γ*, and *TNF-α* mRNA expression levels were significantly increased in NET-added macrophages compared to those in macrophages alone, and these mRNA increases were inhibited by pre-treatment of neutrophils with Cl-amidine (Fig. 5). There was no significant difference in mRNA expression levels between macrophages derived from *Il36rn*^−/−^ mice and those from wild-type mice. Thus, the production of IL-1β, IL-36γ, and TNF-α by macrophages was increased by NET stimulation in vitro.

## Discussion

The results of our study demonstrate that IMQ treatment for 3 consecutive days induced psoriasis-like lesions in *Il36rn*^−/−^ mice (Fig. 1A,B). The severity of the disease is aggravated and accelerated in *Il36rn*^−/−^ mice compared to that in wild-type mice. Many neutrophils infiltrated into the superficial dermis and epidermal thickness and the number of infiltrated MPO-positive neutrophils were significantly increased in *Il36rn*^−/−^ mice compared to those in wild-type mice (Fig. 2A,B,C), which is consistent with previous studies^13^. In addition, real-time RT-PCR analysis showed that *IL-1β*, *IL-17A*, *IL-36γ*, *CXCL1*, and *CXCL2* expression was increased in IMQ-induced psoriasis-like lesions in *Il36rn*^−/−^ mice (Fig. 3). As these results were indicative of the global gene expression occurring in the skin, the source of these genetic changes remains unknown. It has been reported that various immune cells and cytokines are involved in the pathogenesis of IMQ-induced psoriasis-like lesions^15–20^. TLR7 stimulation by IMQ causes the production of IL-23 and IL-36 from DCs^14^ and early IL-23 production is caused by IL-36 signaling in keratinocytes^43^. The αβ T cells or γδ T cells, which are main producers of IL-17 in IMQ-induced psoriasis-like lesions, got activated by IL-23, and secrete IL-17 and IL-22, resulting in the proliferation of keratinocytes^14^. IL-36 derived from DCs stimulates themselves and keratinocytes to further produce IL-36. Additionally, activated keratinocytes produce CXCL1 and CCL20 to facilitate the migration of neutrophils, αβ T cells, and γδ T cells^14^. Although the presence of γδ cells was not demonstrated in *Il36rn*^−/−^ mice, considering previous reports, our results might indicate that stimulation by IL-36 derived from DCs causes the proliferation of keratinocytes and that various chemokines produced by keratinocytes induce the migration of neutrophils, αβ T cells, and γδ T cells. However, the difference in IL-23p19 expression in skin lesions did not reach significance in *Il36rn*^−/−^ mice compared to that in wild-type mice (Fig. 3). It has been reported that not only IL-23 but also IL-1β induces IL-17 production from γδ T cells^44^. As *IL-1β* mRNA levels in skin lesions were significantly elevated in *Il36rn*^−/−^ mice compared to those in wild-type mice (Fig. 3), the increase in IL-17A in *Il36rn*^−/−^ mice would be caused mainly by CCL20 and IL-1β rather than the IL-23/IL-17/IL-22 axis.

*Il36rn*^−/−^ mice showed a significant increase in the number of infiltrated MPO-positive neutrophils (Fig. 2C), which is consistent with the results of previous studies^13^. In addition, the area of NETs was also increased in *Il36rn*^−/−^ mice compared to that in wild-type mice (Fig. 2E). It has been reported that NETs activate keratinocytes and promote the production of LCN2, IL-36γ, CXCL8, and CXCL1^20^. Endogenous neutrophil-derived TLR4 ligands synergize with IL-36, signaling through MyD88 and NF-κB activation, to induce LCN2 and IL-36γ production. In turn, the upregulated LCN2 modulates NET formation and neutrophil migration, enhancing and sustaining the inflammatory response^20^. In accordance with a previous report, IL-36γ and CXCL1 were increased in IMQ-induced psoriasis-like lesions in *Il36rn*^−/−^ mice, abundant with NETs (Fig. 3). Next, we examined the effect of Cl-amidine, which is a pan-PAD inhibitor, in IMQ-induced psoriasis-like lesions of *Il36rn*^−/−^ mice. The administration of Cl-amidine significantly inhibited NET formation in *Il36rn*^−/−^ mice. In addition, PASI scores, the epidermal area, and the number of infiltrated MPO-positive cells were significantly reduced by Cl-amidine administration compared with those measured in vehicle-treated *Il36rn*^−/−^ mice (Fig. 4B and C). Furthermore, Cl-amidine-treated *Il36rn*^−/−^ mice with psoriasis-like lesions showed decreased TNF-α, CXCL1, IL-1β, IL-36γ, and IL-17A expression compared to those in untreated, IMQ-induced *Il36rn*^−/−^ mice. Thus, the inhibition of NET formation improved IMQ-induced psoriasis-like lesions in *Il36rn*^−/−^ mice. Since Cl-amidine is a pan-PAD inhibitor, it is therefore possible that the effect of Cl-amidine on *Il36rn*^−/−^ mice is due to the inhibition of PADs other than PAD4. PAD1 mutations have been associated with the severity of psoriasis in patients^45^, and PAD1 is importantly expressed in skin keratinocytes and hair follicles^46^. Although the effect of Cl-amidine could affect PAD1 in skin keratinocytes and hair follicles, Shao et al. also reports that the administration of Cl-amidine to IMQ-induced psoriasis-like lesions is as effective as DNase I, which breaks down NETs, in scaling, acanthosis, and inflammatory infiltration^20^. As this information is merely comparison of the effect between Cl-amidine and DNase I, it strongly suggests that the inhibition of PAD4 by Cl-amidine suppressed NET formation and improved IMQ-induced psoriasis-like lesions in *Il36rn*^−/−^ mice. Collectively, increased MPO-positive neutrophil infiltration and NET formation in *Il36rn*^−/−^ mice would play a central role in the pathogenesis of IMQ-induced psoriasis-like lesions.

NETs promote macrophage pyroptosis and the production of macrophage-derived Il-1β and TNF-α^32^. In addition, it has been reported that macrophages and monocytes secrete various cytokines such as IL-36^42^. Therefore, we investigated the changes in IL-1β, IL-36γ, and TNF-α production by macrophages upon NET stimulation. *IL-1β*, *IL-36γ*, and *TNF-α* mRNA expression levels were significantly increased in NET-added macrophages compared to those in macrophages alone (Fig. 5). These mRNA increases were inhibited by pre-treatment of Cl-amidine (Fig. 5). These results suggest that various cytokines involved in the pathogenesis of IMQ-induced psoriasis-like lesions would be produced not only by keratinocytes but also by macrophages and that NETs could promote the production of these cytokines.

**Figure 6:**
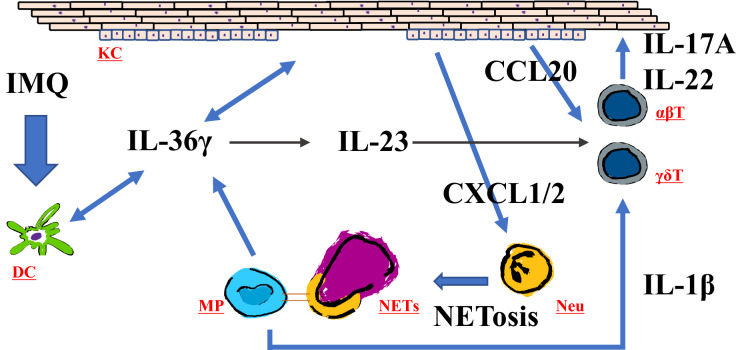
Scheme illustrating pathology of psoriasis-like lesions. Neutrophils cause NETosis, and cytokines, including IL-1β and IL-36γ are secreted by macrophages activated by NETs, and the production of IL-17A from γδ T cells is promoted by IL-1β or CCL20 from imiquimod (IMQ)-induced psoriasis-like lesions. KC: keratinocytes DC: dendritic cells MP: macrophages NETs: neutrophil extracelluar traps Neu: neutrophils. αβT: αβT cells γδT: γδT cells.

Based on our results and previous reports, the mechanisms of IMQ-induced psoriasis-like lesions in *Il36rn*^−/−^ mice are illustrated in Fig. 6. DCs activated by IMQ secrete IL-36γ, causing the proliferation of keratinocytes. CCL20 generated from the keratinocytes mainly enhances the migration of γδ T cells and the secretion of a large amount of IL-17A. Neutrophils can cause NETosis, and cytokines, including IL-1β, IL-36γ, and TNF-α ,are secreted by macrophages activated by NETs; moreover, MyD88/NF-kB activation by IL-36γ and the production of IL-17A from γδ T cells promoted by IL-1β form a pathological condition of IMQ-induced psoriasis-like lesions.

In summary, our results suggest that IL-36Ra loss causes the development of severe psoriasis-like lesions after IMQ treatment for 3 consecutive days by increasing the infiltration of neutrophils into the skin, which is associated with the activation of IL-36R-mediated sustained inflammatory signaling. NET formation also contributes to the development of severe psoriasis-like lesions in *Il36rn*^−/−^ mice. Therefore, NETs may represent promising therapeutic targets for DITRA.

## Methods

### Mice

*Il36rn*^−/−^ mice were generated as previously reported^11^. All experiments were repeated twice using fertile and healthy mice that displayed no evidence of disease or infection. All mice were housed in a pathogen-free barrier establishment and were screened regularly for pathogens. Male C57BL/6 N (Charles River Laboratories, Inc., Wilmington, MA, USA) mice aged 8–12 weeks were used in this study. All studies and procedures were approved by the Animal Care and Use Committee of Fujita Health University (AP16113) and performed in accordance with the National Institutes of Health guide for the care and use of laboratory animals.

### Psoriasis model in mice

The back skin of male wild-type or *Il36rn*^−/−^ mice was shaved using an electric clipper and depilatory cream before treatment. To establish a mouse model of psoriasis, wild-type and *IL36rn*^−/−^ mice were topically administered IMQ cream (62.5 mg Beselna Cream, containing 5% IMQ; kindly gifted by MOCHIDA PHARMACEUTICAL CO., LTD., Tokyo, Japan) on the back skin for 3 consecutive days over an area of 3 × 2 cm (Fig. 1A). In some experiments, Cl-amidine (10 mg/kg/day, Cayman Chemical Company, Ann Arbor, MI, USA), a PAD4 inhibitor, was subcutaneously injected.

### PASI assessment

The severity of skin lesions was graded according to PASI, which assesses skin erythema, scaling, and thickness. PASI scores were 0–4, as follows: 0, none; 1, slight; 2, moderate; 3, marked; 4, severe^35^. The mice in each group were assessed on day 4.

### Histological assessment

Mice were sacrificed via cervical dislocation, and the fresh back skin of each mouse was harvested. Fresh mouse skin samples were fixed in 4% paraformaldehyde solution for 24 h, dehydrated, and embedded in paraffin. Paraffin sections were stained with hematoxylin and eosin. Epidermal thickness was evaluated by measuring the area of the epidermis. The area of the epidermis within a distance of 10 mm was measured using ImageJ software (version 1.53; NIH, Bethesda, MD, USA, https://imagej.nih.gov/ij/)^47^. Neutrophil infiltration was assessed by counting the number of neutrophils in nine high-power fields per section. Each section was examined independently by two investigators in a blinded manner, and the means of these measurements were used for analysis.

### Tissue immunofluorescence and immunohistochemical staining

For immunofluorescence staining, the skin sections were fixed, stained, and imaged via confocal microscopy. Embedded tissues were cut into 5-µm sections and washed using phosphate-buffered saline (PBS). These sections were treated with blocking solution (normal donkey serum diluted 1:19 in buffer: PBS + 1% bovine serum albumin) for 30 min at 27 °C. To evaluate the location of NETs, mouse skin tissues were incubated with specific primary antibodies for MPO (1:100, Research and Diagnostic Systems, Inc., Minneapolis, MN, USA) and citrullinated-histone H3 (1:250, Abcam, Cambridge, UK) for 2 h at 27 °C. The sections were then washed with PBS and incubated with secondary antibodies (donkey anti-goat immunoglobulin G (IgG), Novus Biologicals, Littleton, CO, USA; Alexa Fluor 488, Thermo Fisher SCIENTIFIC, Waltham, MA, USA; donkey anti-rabbit IgG, Novus Biologicals; and Alexa Fluor 647, Thermo Fisher SCIENTIFIC) for 30 min at 27 °C. The sections were then enclosed in Fluoroshield mounting medium with 4′,6-diamidino-2-phenylindole (Abcam). Confocal images were acquired using an Olympus Fluoview 1000 microscope (Olympus Life Science, Tokyo, Japan) with a PlanApo N (× 40 with and without 2.5 digital zoom). Immunofluorescence of CD11c (1:200, Abcam) was also performed and Alexa Fluor 647 was used as a secondary antibody.

For immunohistochemistry of mouse samples, 6-μm-thick sections of paraffin-embedded tissues were cut, deparaffinized in xylene, and rehydrated in phosphate buffered saline (PBS). Deparaffinized sections were treated with endogenous peroxidase blocking solution (horse serum diluted 1:1 in buffer: PBS + bovine serum albumin 1%) for 15 min at room temperature. Sections were then incubated overnight at 4 °C with rabbit monoclonal antibodies (mAb) specific for CD3 (1:500, Cell Signaling Technology, Inc., Tokyo, Japan), and F4/80 (1:800, Cell Signaling Technology, Inc.). Sections were then washed in PBS buffer and biotin-conjugated secondary antibodies were applied, followed by incubation with VECTASTAIN Elite ABC Kit (Vector Laboratories, Inc., Burlingame, CA, USA) for 30 min at room temperature and three washes with PBS for 15 min each. Peroxidase activity was observed using an ImmPACT DAB Substrate Kit (Vector Laboratories, Inc.) and samples were counterstained with hematoxylin. For a negative control, primary antibody was not added to the sections.

### RNA extraction and real-time RT-PCR

Qiagen RNeasy spin columns (QIAGEN, Valencia, CA, USA) was used to extract total RNA from the mouse back skin tissue samples^11^. The total RNA was reverse-transcribed into cDNA using a Prime Script RT Reagent Kit (Takara Bio Inc., Otsu, Japan). Expression levels of genes encoding IL-1β, IL-6, IL-17A, IL-36γ, CCL4, CCL5, CXCL1, CXCL2, TNF-α, and IL-23p19 were measured via real-time RT-PCR using a Light Cycler System (F. Hoffman-La Roche, Ltd, Basel, Switzerland)^11^. PCR samples were prepared in microcapillary tubes as 20-µL reactions containing diluted cDNA solution (2.0 µL), and the PCR program was performed according to the manufacturer’s instructions. Glyceraldehyde-3-phosphate dehydrogenase (*GAPDH*) expression was used as a standard against which the relative mRNA expression levels of different target genes were calculated using the 2^*−*∆∆Ct^ method. Primer sequences used for each gene were provided with the pre-verified Primetime qPCR Assay (Integrated DNA Technologies, Inc., Coralville, IA, USA)^12^.

### In vitro experiments

#### Collection of peritoneal cells from the peritoneal cavities of mice

Mice were intraperitoneally injected with cold PBS (10 mL). The abdomen was massaged to suspend the cells, and 8 mL PBS was collected. The cells obtained were centrifuged at 500 × *g* at 4 °C for 10 min. Red blood cell lysis buffer was added, and the cells were again centrifuged at 500 × *g* at 4 °C after incubation at 4 °C for 10 min to obtain the peritoneal cells^48^.

#### Collection of neutrophils from the peritoneal cavities of mice

Neutrophils were collected from the peritoneal cavities of mice using a Neutrophil Isolation Kit (Cayman Chemical Company) according to the manufacturer’s protocol. Casein (7.5%, 1 mL) was injected into the peritoneal cavity of mice followed by neutrophil isolation medium (5 mL, PBS + 1% BSA) 24 h later. The abdomen was massaged to suspend the cells, and the medium (4 mL) was collected. The fluid from the peritoneal cavity was slowly layered onto 63% Percoll solution, which was then centrifuged at 1,000 × *g* at 27 °C for 20 min. The Percoll solution was aspirated, and PBS with BSA (9 mL) was added. The samples were centrifuged at 500 × *g* at 27 °C for 5 min. Red blood lysis buffer (5 mL) was added, and the samples were centrifuged at 500 × *g* at 27 °C for 10 min after incubation at 27 °C for 10 min to obtain the mouse neutrophils.

#### NET generation

For NET generation, 1 × 10^6^ neutrophils were seeded into 24-well plates in serum-free Dulbecco’s modified Eagle’s medium (DMEM) and then stimulated to release NETs by adding phorbol 12-myristate 13-acetate (50 nM; Sigma-Aldrich Co. LLC., St Louis, MO, USA) for 4 h at 37 °C. The medium was removed, and the cell layer was carefully washed with PBS (2 mL). The PBS was collected after vigorous agitation and centrifuged at 500 × *g* at 4 °C for 10 min. Cell-free NET structures were then collected in the supernatant phases^20^. Some neutrophils were pretreated with Cl-amidine (10 μM; Cayman Chemical Company) before NET generation for 2 h at 37 °C^49^.

#### Macrophage culture

Peritoneal cells (mixture of macrophages, B cells, and T cells, 0.5 × 10^6^) were cultured in 24-well plates in serum-free DMEM. After 24 h, non-adherent cells (B cells and T cells) were removed by gently washing three times with warm PBS^48^. In some experiments, isolated macrophages were co-cultured with NETs originated from 0.5 × 10^6^ neutrophils. After 12 h of culture, total RNA was extracted and subsequently reverse-transcribed.

### Statistical analysis

Data were analyzed using a GRAPHPAD PRISM software (version 7, Graph Pad Software, La Jolla, CA, USA, https://www.graphpad.com/scientific-software/prism/) and presented as means ± standard deviations. For comparisons between groups, Mann–Whitney *U* tests and one-way analysis of variance were used. Values of *p* < 0.05 were considered to indicate statistical significance.

## Supplementary information

Supplementary Information 1.

Supplementary Information 2.

Supplementary Information 3.

## Data Availability

The datasets generated and/or analyzed during the current study are available from the corresponding author upon reasonable request.
