# Supplementary material for: Neutrophil extracellular traps are induced in a psoriasis model of interleukin-36 receptor antagonist-deficient mice
Source: Sci Rep. 2020 Nov 19;10:20149. doi: 10.1038/s41598-020-76864-y (PMC7678853; doi:10.1038/s41598-020-76864-y)
Supplement: Supplementary file 1 — Supplementary Information 1. [file 41598_2020_76864_MOESM1_ESM.pptx]

## Slide 1
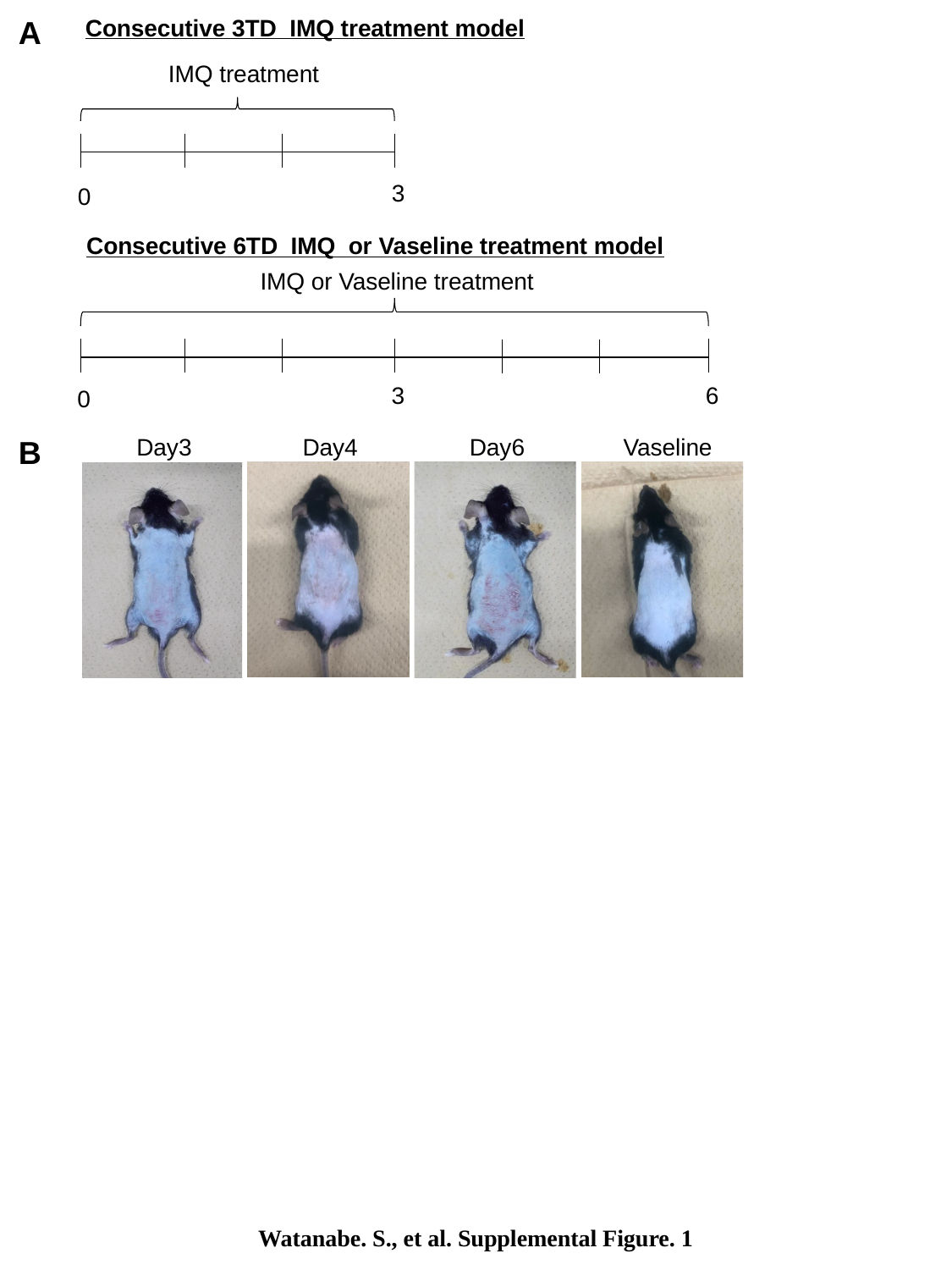

A
Consecutive 3TD IMQ treatment model
IMQ treatment
3
0
Consecutive 6TD IMQ or Vaseline treatment model
IMQ or Vaseline treatment
3
6
0
B
Day3
Day4
Day6
Vaseline
Watanabe. S., et al. Supplemental Figure. 1
