# Supplementary material for: Neutrophil extracellular traps are induced in a psoriasis model of interleukin-36 receptor antagonist-deficient mice
Source: Sci Rep. 2020 Nov 19;10:20149. doi: 10.1038/s41598-020-76864-y (PMC7678853; doi:10.1038/s41598-020-76864-y)
Supplement: Supplementary file 2 — Supplementary Information 2. [file 41598_2020_76864_MOESM2_ESM.pptx]

## Slide 1
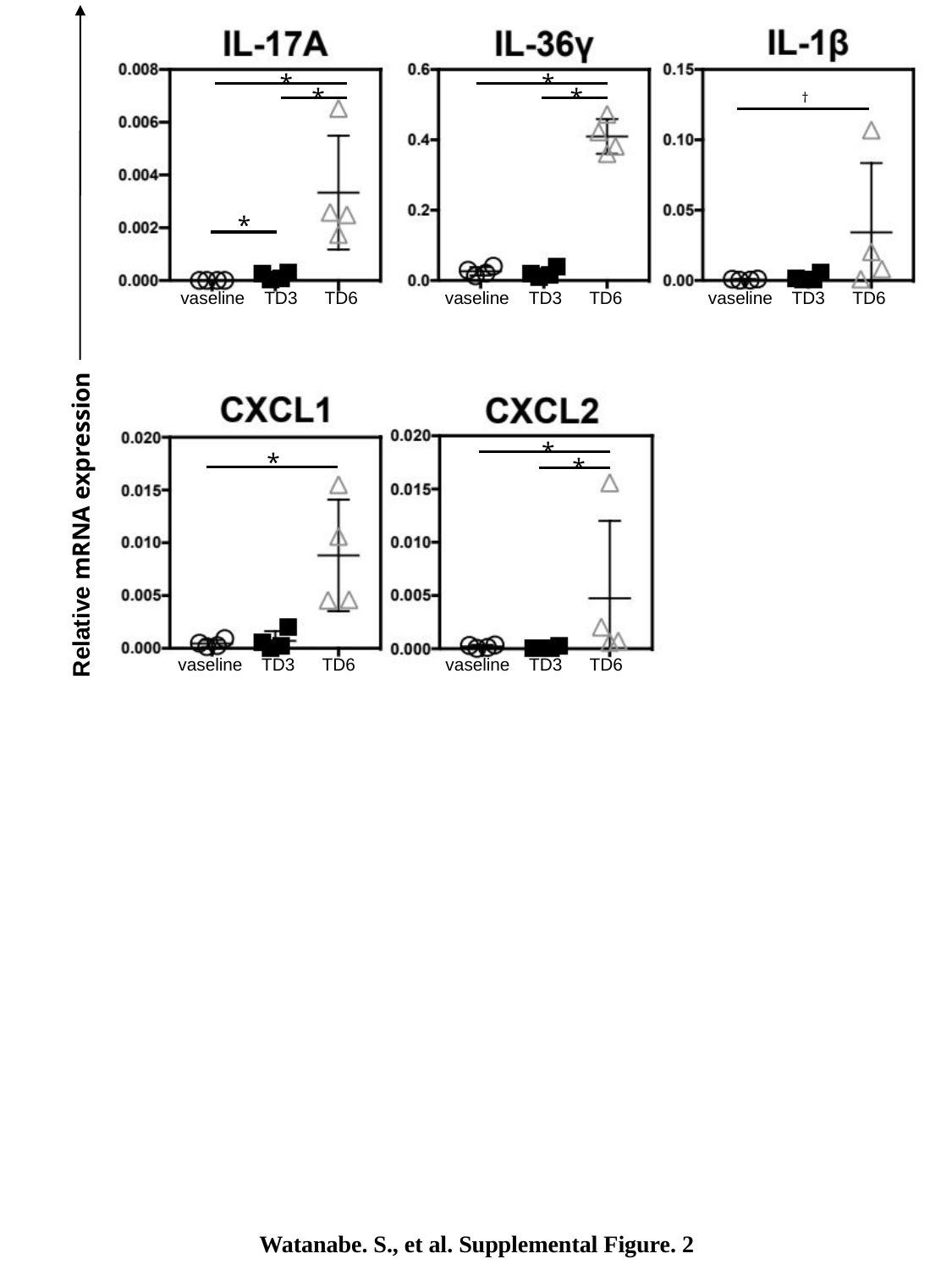

*
*
*
*
†
*
vaseline
TD3
TD6
vaseline
TD3
TD6
vaseline
TD3
TD6
*
*
*
Relative mRNA expression
vaseline
TD3
TD6
vaseline
TD3
TD6
Watanabe. S., et al. Supplemental Figure. 2
