# Supplementary material for: Neutrophil extracellular traps are induced in a psoriasis model of interleukin-36 receptor antagonist-deficient mice
Source: Sci Rep. 2020 Nov 19;10:20149. doi: 10.1038/s41598-020-76864-y (PMC7678853; doi:10.1038/s41598-020-76864-y)
Supplement: Supplementary file 3 — Supplementary Information 3. [file 41598_2020_76864_MOESM3_ESM.docx]

**Supplemental materials for**

**Neutrophil extracellular traps are induced in a psoriasis model of interleukin-36 receptor antagonist-deficient mice**

Soichiro Watanabe^1^, Yohei Iwata^1^, Hidehiko Fukushima^1^, Kenta Saito^1^,

Yoshihito Tanaka^1^, Yurie Hasegawa^1^, Masashi Akiyama^2^, Kazumitsu Sugiura^1*^

^1^Department of Dermatology, Fujita Health University School of Medicine, 1-98 Kutsukake-cho, Toyoake, Aichi 470-1192, Japan

^2^Department of Dermatology, Nagoya University Graduate School of Medicine, 65 Tsurumai-cho, Showa-ku, Nagoya, Aichi 466-8560, Japan

**Supplemental methods**

We considered that the consecutive application of IMQ for 3 days was not enough to increase mRNA expression in WT mice. Therefore, to test this possibility, we performed following additional experiments. Wild-type mice treated with IMQ for consecutive 6 days was compared with wild-type mice treated with IMQ for consecutive 3 days (supplemental figure. 1-a). As a control, some of mice were treated with Vaseline for consecutive 6 days. The total RNA was reverse-transcribed into cDNA using a Prime Script RT Reagent Kit (Takara Bio, Inc., Shiga, Japan). Expression levels of genes encoding IL-17A, IL-1β, IL-36γ, CXCL1, and CXCL2 were measured via real-time RT-PCR using a LightCycler® (Roche, Basel, Switzerland).

**Results**

Erythema, scaling, and infiltration were significantly increased in the mice for 6 consecutive days IMQ treatment in comparison to the mice for 3 consecutive days IMQ treatment (supplemental figure. 1-b). IL-17A, IL-36γ, and CXCL2 levels were significantly increased in the mice for 6 consecutive days IMQ treatment in comparison to the mice for 3 consecutive days IMQ treatment (n = 4, **p* <0.05, supplemental figure. 2).
